# Long-term follow-up and survival of delayed total hip arthroplasty following acetabular fracture: a matched cohort study of 552 cases from the Norwegian Arthroplasty Register

**DOI:** 10.1177/11207000231212884

**Published:** 2023-11-21

**Authors:** Ragnhild Loven Kirkeboe, Lars Nordsletten, Jan Erik Madsen, Eva Dybvik, Stein Atle Lie, Geir Hallan, John Clarke-Jenssen

**Affiliations:** 1 Division of Orthopaedic Surgery, Oslo University Hospital, Oslo, Norway; 2 Institute of Clinical Medicine, University of Oslo, Oslo, Norway; 3 The Norwegian Arthroplasty Register, Department of Orthopaedic Surgery, Haukeland University Hospital, Bergen, Hordaland, Norway; 4 Department of Clinical Dentistry, University of Bergen, Norway; 5 Department of Clinical Medicine, University of Bergen, Norway

**Keywords:** Acetabulum, post-traumatic arthritis, secondary total hip arthroplasty

## Abstract

**Background:**

Operative treatment of acetabular fractures generally yields good results, but several authors report up to 15–20% of patients developing post-traumatic osteoarthritis (OA). Previous studies have shown that total hip arthroplasty (THA) following post-traumatic OA have inferior results compared to THA for primary OA. The aim of this study was to report on long-term outcome of THA following acetabular fracture, compared to primary OA.

**Materials and methods:**

We performed a matched cohort study with data from the Norwegian Arthroplasty Register (NAR). All patients receiving THA following an acetabular fracture between 1987 and 2018 were identified. A 3:1 matched cohort consisting of patients treated for primary OA with THA was selected using propensity scores and matched for age, gender and year of surgery. Survival analysis was performed with revision of any cause as endpoint. Cox regression was used to identify factors associated with risk of revision surgery.

**Results:**

552 cases were identified, 397 men and 155 women. Mean age was 58.8 (11–91) years. 224 had previously been operated for the acetabular fracture, 328 had been treated non-operatively. Mean follow up time was 8.7 (1–29) years. Implant survival at 10 years was 79.7% (75.6–83.3) and at 20 years 62.4% (55.5–69.3). The hazard ratio for revision was 1.38 (1.07–1.77, *p* < 0.001) compared to the OA cohort, regardless of operative or non-operative treatment of the index acetabular fracture. Uncemented acetabular components had an increased risk of revision with hazard ratio for revision 1.61 (*p* = 0.012).

**Conclusions:**

THA following an acetabular fracture can be performed with acceptable results regarding implant survival, however, we report an increased risk for revision when compared to primary OA. Our results indicate that previous operative fracture treatment does not increase the risk for THA revision compared to cases treated non-operatively.

## Introduction

Acetabular fractures are serious injuries that require accurate reduction and fixation in order to secure a pain free hip joint. Current treatment for most displaced fractures is open reduction and internal fixation (ORIF). Generally, the results are good.^1–4^ Despite adequate fracture treatment, post-traumatic osteoarthritis (OA) has been reported to afflict between 11 and 67%.^4–7^ In later years, the incidence of geriatric acetabular fractures has increased.^3^ These fractures are more comminuted and difficult to reconstruct,^8^ and this may lead to an increasing number of patients developing post-traumatic OA.

The most common treatment for symptomatic post-traumatic OA is total hip arthroplasty (THA) and up to 20% of all patients may require a THA within 20 years after an acetabular fracture.^1,3,5,9^

Whilst results for THA due to primary OA are excellent, inferior results have been reported for THA after an acetabular fracture, regarding both implant survival and complications.^10–15^ In a retrospective case-control study with uncemented implants, 10-year survivorship of THA after acetabular fracture was 70% versus 90% for THA due to primary OA. The same study also demonstrated an increased risk of complications.^10^ There is an ongoing debate whether cemented or uncemented implants should be preferred. Scott et al.^16^ reported 92% 10-year survival of cemented THA in a series of 49 patients. Bellabarba et al.^17^ reported excellent results with a 10-year survival of 97% for uncemented THR, in a rather small series. The present study is a matched-cohort study, based on data from the Norwegian Arthroplasty Register (NAR), and thus able to report long-term results on THA following acetabular fracture in large number of cases.

The aim of this study was to report on long-term survival of THA following an acetabular fracture compared to THA due to primary OA. We also aimed to identify factors that were associated with an increased risk for revision in the study group.

## Material and methods

We performed a register-based propensity matched cohort study. Since 1987, THAs performed in Norway, including revision surgeries, have been registered in the NAR. Surgeons report a baseline set of data, including the indication of surgery, surgical approach, type of implant and implant fixation. The completeness of NAR is good with more than 97% of primary surgeries and 93% of revisions reported annually.^18^

All cases of THA following an acetabular fracture were identified and included from the start of the register 01.09.87 until 31.12.18. Patients treated with THA for acute acetabular fracture (*n* *=* 83), arthritis following traumatic hip dislocation (*n* *=* 133) and post-traumatic avascular necrosis of the femoral head (*n* *=* 43) were excluded, as there was no information available confirming that these patients were also treated for acetabular fracture.

552 cases were identified and included, and constituted the study group. A propensity score was calculated and used to match cases 1:3 with patients who received THA due to primary OA.

The primary outcome was implant survivorship, with revision of any implant component for any cause as the endpoint. Secondary outcome measures were specific causes for revision surgery such as aseptic loosening, infection, recurrent dislocation, and an evaluation of factors that may influence implant survival, such as the year of surgery, age, gender, fixation of the implant and operative versus non-operative treatment of the index acetabular fracture.

### Statistics

The statistical analyses were performed using SPSS (version 25, IBM Corp, New York, USA).

For each case, a propensity score was calculated, and three matches were identified from all patients in the registry. The groups were matched on age, gender and year of surgery. To secure accuracy of the propensity score matching, we also did a trial case-control matching with a limited number of controls from the registry. Accuracy when using the propensity score was shown to be better, hence this method was preferred.

Survival analysis was done with the Kaplan Meier method with revision of any cause as endpoint, and differences in survivorship between cases and controls were tested with the Log-Rank test. Cox regression models were used to explore factors that could be associated with an increased risk of revision, and to estimate hazard ratios. Statistical significance was set at *p* < 0.05.

For ethical approval, the study was approved through the NAR. The NAR has a license from the Norwegian Data Inspectorate (reference number: 03/00058-20/CGN; date of issue: latest license, 15 September 2014).

## Results

552 cases of THA following an acetabular fracture were identified. There were 397 men and 155 women with a mean age of 58.8 (11–90) years at the time of THA in the study group. The mean follow-up time was 8.7 (1–29) years after THA. Mean age in the matched cohort was 66 years, and there were 901 men and 753 women. The differences in age and gender between the 2 groups were statistically significant, however as all patients with previous acetabular fracture were included, this was accepted. The total propensity score did not differ significantly.

224 cases with a previous acetabular fracture had initially been treated operatively and 328 had been treated non-operatively for the index acetabular fracture. No further details regarding the treatment or the classification of the fractures were attainable, as this is not included in the NAR database. 159 patients had the THA done in centres that provide operative pelvic fracture treatment; 3 university hospitals have this service in our country.

### Implants

299 hips had cemented and 249 had uncemented acetabular cups, and 265 had cemented and 279 had uncemented femoral stems in the study group. 51 different acetabular components and 42 different femoral components were used, reflecting that cases were treated in a great variety of institutions over a long period of time. Tables 1 and 2 constitute a list of the 5 most commonly used acetabular and femoral implants.

**Table 1.** The 5 most commonly used acetabular cups in the study group, the control group and in the complete arthroplasty register.

Study group	*n*	Percent (%)
Charnley Ogee*	80	14.5
Marathon*	65	11.8
Elite*	42	7.6
Trabecular Metal	30	5.4
Reflection uncemented	27	4.9
**Control group**		
Charnley Ogee*	651	39.3
Exeter Contemporary*	154	9.3
Spectron, spectron EF*	101	6.1
Titan*	88	5.3
Tropic	70	4.2
**Complete register - primary OA**		
Charnley Ogee*	32143	21.4
Marathon*	20666	13.8
Reflection cemented all poly*	11270	7.5
Exeter Contemporaty*	10721	7.1
Exeter X3 rimfit*	8627	5.8

OA, osteoarthritis.

* Cemented cup.

**Table 2.** The 5 most commonly used femoral stems in the study group with, in the control group and in the complete arthroplasty registry.

Study group	*n*	Percent (%)
Corail	158	28.6
Charnley*	89	16.2
Exeter*	78	14.2
Profile	18	3.3
Filler	18	3.3
**Control group**		
Charnley*	677	40.9
Corail	257	15.5
Exeter*	187	11.3
Titan*	115	6.9
ITH*	70	4.2
**Complete register – primary OA**		
Corail	40698	27.2
Charnley*	31688	21.2
Exeter*	25223	16.9
Titan*	9016	6
Spectron*	8533	5.7

OA, osteoarthritis.

* Cemented stem.

### Survivorship

The risk for revision was increased in the study group when compared to the matched cohort with a hazard ratio (HR) for revision 1.38 (1.07–1.77, *p* *<* 0.001).

Kaplan-Meier implant survivorship for the study group at 10 years was 79.7% (95% CI, 75.6–83.3%), and at 20 years survival rate was 62.4% (95% CI, 55.5–69.3%) (Table 3).

**Table 3.** Cumulative Kaplan-Meier survival of THR in the study group with 95% CI and the number of cases left at risk at time of follow-up.

	Survival, % (95% CI)	Left at risk
5-year	89.4 (86.7–92.1)	332
10-year	79.7 (75.6–83.8)	193
15-year	70.1 (64.6–75.6)	103
20-year	62.4 (55.5–69.3)	58

CI, confidence interval.

**Figure 1.**
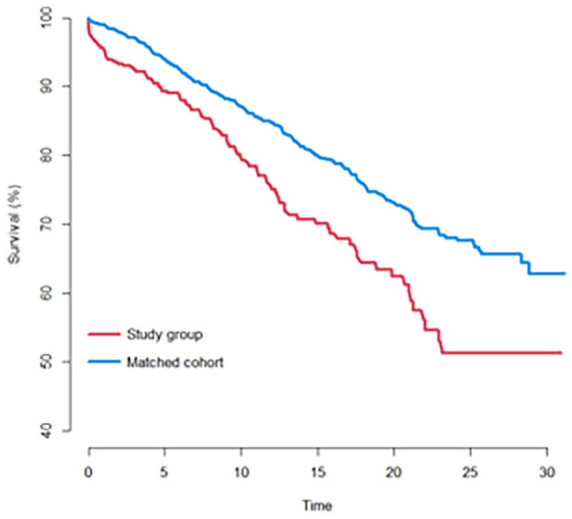
Cumulative survival of the total hip arthroplasty over time with years to revision as failure. Red (lower line on graph): Study group of cases previously treated for acetabular fracture. Blue (upper line on graph): Matched cohort of primary OA cases.

Figure 1 shows a Kaplan-Meier plot of cumulative survival.

**Figure 2.**
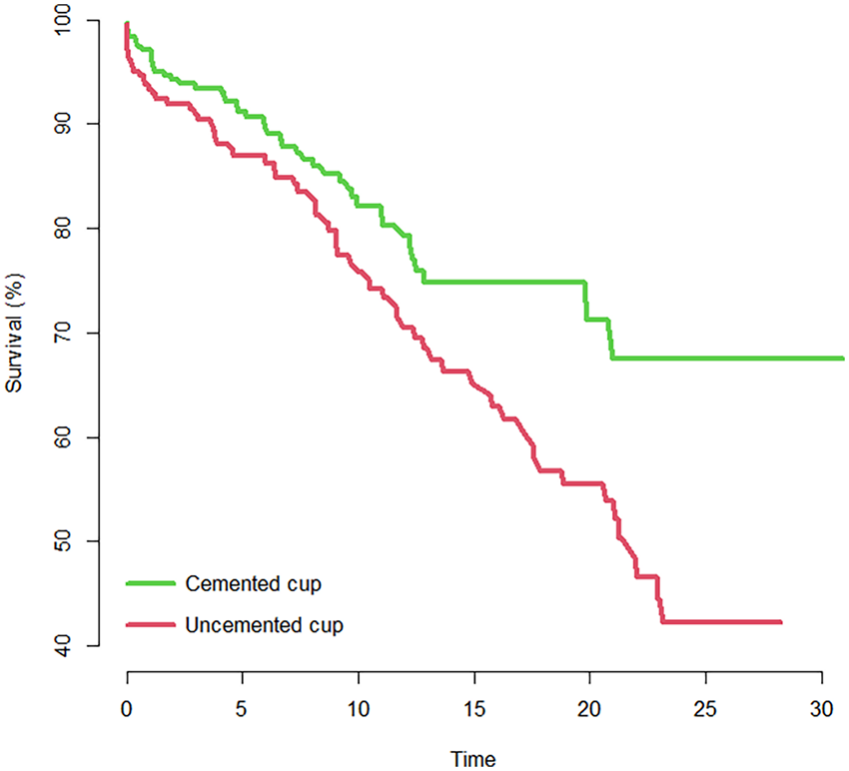
Kaplan Meier survival plot showing cumulative survival of acetabular cups in the study group, with years to revision as end-point. Cemented cups in green (upper line on graph) and uncemented cups in red (lower line on graph).

Uncemented cup fixation and cemented stem fixation was associated with and increased risk of THA revision; HR 1.61 (1.10–2.42, *p* *=* 0.012) and HR 1.62 (1.10–2.39; *p* *=* 0.013) (Figure 2).

### Causes for revision

There were 116 (21%) revisions for any reason in the study group. In the control group, there were 181 revisions; 10.9% of the 1654 cases.

The most frequent reason for revision in both groups was aseptic loosening of the acetabular cup, followed by aseptic loosening of the femoral stem and deep infection (Table 4).

**Table 4.** Causes for revision in the study group and in the control group.

Cause for revision	Study group, *n* (%)	Control group, *n* (%)
Aseptic loosening cup	38 (32)	64 (35)
Aseptic loosening stem	23 (19)	46 (25)
Deep infection	22 (18)	27 (14)
Recurrent dislocation	16 (13)	15 (8)
Other	17 (14)	29 (16)
Total	116	181

As multiple reasons for revision can be listed for the same procedure in the register, the total number of indications exceeds the number of revisions performed.

### Risk factors

We attempted to identify factors that influenced implant longevity in the study group. Gender and certain age groups had an increased risk of revision; male gender with a HR 1.86 (95% CI, 1.12–3.1 and *p* *=* 0.017), age <45 years HR 2.83 (95% CI, 1.09–7.37; *p* *=* 0.033) and age 55–65 years HR 2.79 (95% CI, 1.08–7.27; *p* *=* 0.035). Previous operative or non-operative treatment of the index acetabular fracture was not found to be a significant risk factor (*p* *=* 0.79).

## Discussion

In this study, we report long-term survivorship of THA for OA secondary to an acetabular fracture. We found a 10-year survival of 79% and a 20-year survival of 61%, and this was poorer than for patients with primary OA. Uncemented cup fixation, male gender and younger age was associated with an increased risk for revision in the study group.

In previous studies, THA subsequent to acetabular fractures has achieved inferior results compared to primary OA. A retrospective case-control study of 74 patients, reported 70% implant survival after 10 years in a group with previous acetabular fracture compared to 90% in the primary OA group.^10^ A systematic review of 422 delayed THAs in patients with previous acetabular fracture reported 10-year cup survival of 76% and stem survival of 85% as well as increased complication rates.^9^ With regard to long-term THA survival, our results seem to confirm previous findings.^13^

It is unclear whether initial operative treatment of acetabular fractures affect later THA longevity. Morison et al.^10^ did not find such a difference in outcome, in congruence with other authors.^12,19^ Some have argued that previous ORIF reduces residual acetabular bone defects and that reconstructing the anatomy facilitates later THA,^20^ whereas others claim that previous surgical treatment leads to prolonged THA operating times and increased blood loss.^17,19^ Retained hardware may also conflict with optimal implant positioning, as well as increase the risk of infection.^21^ We could not find any differences in implant survival between cases treated operatively or non-operatively for their index fracture.

Aseptic loosening of the acetabular cup is a major concern in post-trauma cases. It is unclear whether cemented or uncemented cup perform better; some modern, un-cemented cups have demonstrated promising resu-lts,^10,12,15,17,19,20,22^ whereas other authors have reported good results with cemented acetabular cups.^16,23^ We observed that the most common indication for revision was aseptic loosening of the acetabular component. We also report an increased risk for revision in uncemented acetabular cups (HR 1.61, *p* = 0.012) when compared to cemented acetabular cups. Historically, uncemented metal-backed modular cups have had poor results in our country, mainly due to UHMWPE wear and osteolysis.^24,25^ Our study group was treated over a long time-period, thus these poorly performing older cup designs may have affected our findings. Over 50 different cup designs were used in the study group and more than 20 different cup designs were used in less than 5 cases. Our study is underpowered to only study contemporary implants. Hence, making a clear recommendation on cup fixation is difficult.

We observed that cemented femoral stems had an increased risk for revision (HR 1.62, *p* = 0.013). The 2 most commonly used cemented femoral stems have had inferior results to the most commonly used uncemented stem in this age-group,^26,27^ thus, as with the acetabular cups, our results are affected by the respective implants used. Some femoral stems may have been revised to simplify exposure of the acetabulum during revision, but these cases cannot be identified in the register.

Previous surgeries of the hip increase the risk of complications after THA, such as infections and dislocations.^28,29^ Revision rate due to infection after primary THA is generally around 1%.^30,31^ Our results reveal that 3.9% of cases were revised due to deep infections in the study group, versus 1.6% infections in the matched cohort. This increased infection risk is well-known,^7,14–16,21^ and a systematic review including 422 delayed THAs reported an infection rate as high as 5.6%.^9^ However, in the study group we could not find an increased risk of infection in cases treated operatively for a previous acetabular fracture compared to cases treated non-operatively.

The current study has several weaknesses. We were not able to provide information regarding acetabular fracture classification or chosen operative treatment of the index acetabular fracture, as this is not available in the NAR. We were not able to include functional outcome measures, as these were not registered in the NAR at the time of the study. Also, only patients with revision surgery were reported as failures to the register and a proportion of patients may have suffered a clinical and/or radiological failure without having been revised. There is, however, no obvious reason to believe that these patients are unequally distributed between the study group and the matched cohort.

The present study is, to our knowledge, the largest report on THA following an acetabular fracture. Utilising register data, we were able to report on 20-year THA survivorship. Register-based studies are appropriate when following a patient cohort with rare diseases or conditions, with a long time-period between exposure and the incident to be studied, as is the case with post-traumatic OA following acetabular fractures.^32^

## Conclusion

This is, to our knowledge, the largest study on THAs following acetabular fractures with long-term follow up. Our findings confirm that post-traumatic OA following an acetabular fracture can be treated with THA with acceptable long-term results. There is, however, a significantly increased risk of revision when compared with THA due to primary OA, especially for uncemented acetabular cups, though these results should be interpreted carefully.
